# Back to the Future: Repurposing Metformin, a Metabolically Active Drug, to Treat Mild-to-Moderate Ulcerative Colitis

**DOI:** 10.3390/metabo16040238

**Published:** 2026-03-31

**Authors:** Elisabetta Antonelli, Alessandra Dell’Era, Giovanni Maconi, Gabrio Bassotti

**Affiliations:** 1 Gastroenterology and Endoscopy Unit, Santa Maria della Misericordia Hospital, 06156 Perugia, Italy; elisabetta.antonelli@ospedale.perugia.it; 2 Gastroenterology Unit, Department of Biomedical and Clinical Sciences, “Luigi Sacco” Hospital, University of Milano, 20157 Milan, Italy; alessandra.dellera@unimi.it (A.D.); giovanni.maconi@unimi.it (G.M.); 3 Gastroenterology, Hepatology, and Digestive Endoscopy Section, Department of Medicine, University of Perugia, 06156 Perugia, Italy

**Keywords:** inflammatory bowel disease, metformin, repurposing drug, ulcerative colitis, treatment

## Abstract

The incidence of ulcerative colitis, an entity belonging to the inflammatory bowel diseases, is rising worldwide. Due to the unpredictable nature of its clinical flares and the need for chronic treatment, ulcerative colitis has a huge burden of psychological impairment and reduced quality of life. Although several treatments are available to manage patients with ulcerative colitis, their efficacy is unpredictable, and long-term remission is often difficult to achieve. Moreover, the more recent and expensive drugs are not easily available in many countries. These facts have prompted the research in this field to focus on finding additional treatments for such patients. Among the putative repurposing drugs, metformin, an oral antidiabetic agent used for over seventy years, seems to represent a good candidate. While randomized controlled trials suggest that metformin, as an adjunct to conventional treatments, may improve clinical outcomes in mild-to-moderate ulcerative colitis, population-based observational and genetic studies offer mixed signals regarding its role in long-term disease modification or primary prevention. This, together with the wide availability and the low cost of metformin, might represent a good example of repurposing, as detailed in the present review. However, human mechanistic validation remains limited, underscoring the need for further research.

## 1. Introduction

Ulcerative colitis (UC) is a chronic, relapsing inflammatory bowel disease characterized by a dysregulated immune response to the gut microbiota [1,2], manifesting as inflammation chiefly limited to the colon and rectum [3]. The incidence of UC is rising worldwide, both in developed countries and in emerging regions [4]. The unpredictable nature of disease flares and chronic medication use leads to substantial psychological burden and reduced quality of life [5], making UC a considerable healthcare issue that requires enhanced treatment approaches [6].

The current therapeutic drugs for ulcerative colitis (UC) include aminosalicylates, corticosteroids, immunosuppressants, biological agents, and small molecules [6,7]. While these treatments have improved patient outcomes, they exhibit variable efficacy in the clinical picture and in the maintenance of long-term remission. Furthermore, these drugs can induce significant adverse effects that limit their tolerability [6]. Consequently, research efforts have focused on the development of new pharmacological agents.

Metformin is a key medication for the pharmacological treatment of type 2 diabetes [8,9]. It has been used for diabetes in Europe since the late 1950s, and many guidelines consider metformin, unless contraindicated or not tolerated, as the first-line therapy in the management of type 2 diabetes. Metformin is effective in lowering glucose [10] and preventing the microvascular and macrovascular complications of diabetes. It is a widely available, well-tolerated, and safe drug, and it has a very low cost [8,10]. Beyond its canonical glucose-lowering effects, metformin possesses pleiotropic anti-inflammatory properties that extend across multiple biological systems relevant to UC pathogenesis [11]. For these reasons, metformin is a compelling candidate for being repurposed as a drug for UC treatment.

## 2. Search Strategy

### 2.1. Search Strategy and Data Sources

A comprehensive systematic literature search was performed across Medline (PubMed) and the Science Citation Index (Web of Science) to identify studies evaluating the therapeutic role of metformin in IBD. The search spanned from January 1980 to January 2026. The search utilized various combinations of the following keywords: “*disease remission*”, “*inflammatory bowel disease*”, “*metformin*”, “*quiescent disease*”, and “*ulcerative colitis*”. Logic was refined using the Boolean operators AND, OR, and NOT to ensure the inclusion of all relevant studies while excluding extraneous data.

### 2.2. Study Selection and Inclusion Criteria

The primary search was limited to articles published in the English language; however, a manual search of the institutional library was conducted to identify relevant non-English language journals and historical records published prior to 1980. Studies were eligible for inclusion if they reported clinical outcomes (disease activity indices, endoscopic scores, or inflammatory markers) in patients with IBD or provided significant mechanistic insights via preclinical models.

## 3. Preclinical Studies: Proposed Mechanisms of Action

Evidence from cell-based systems and animal models suggests that metformin may modulate several key inflammatory pathways in UC, including T cell differentiation, toll-like receptor signaling, and nuclear factor-kappa B (NF-κB) activation. Preclinical data also indicate that metformin may influence gut microbiota composition [12]. Furthermore, experimental studies suggest that metformin could reduce pro-inflammatory cytokines and chemokines in models of UC [13], potentially contributing to protection against intestinal barrier dysfunction. It should be noted, however, that these observations derive exclusively from in vitro and animal studies, and their direct applicability to human UC remains to be established.

***Molecular pathway modulation:*** In vitro and murine studies suggest that metformin may activate AMP-activated protein kinase (AMPK) and, via this mechanism, suppress mTOR, NF-κB and JNK signaling; reduce STAT3 phosphorylation; and lower the expression of endoplasmic reticulum (ER) stress-related proteins such as GRP78, CHOP, and caspase-12, collectively leading to reduced epithelial apoptosis and attenuation of inflammatory signaling in experimental settings [13,14,15,16]. The potential therapeutic relevance of these pathways in UC is suggested, though not yet proven in humans, by a convergence of preclinical findings. Specifically, AMPK is a central cellular energy sensor that has been shown, in animal models and cell lines, to drive downstream anti-inflammatory cascades, including suppression of mTOR, inhibition of NF-κB phosphorylation, and reduction in c-Jun N-terminal kinase (JNK) activity events associated with reduced intestinal inflammation in experimental models [14,15]. In vitro data further indicate that metformin may suppress ER stress-induced apoptosis in intestinal epithelial cells by reducing the expression of GRP78, CHOP, and caspase-12 [16]. Additionally, preclinical studies report that metformin inhibits the SPHK1/S1P signaling pathway, reduces myeloperoxidase activity and oxidative stress markers, and suppresses STAT3 phosphorylation in experimental models [13]. Taken together, these preclinical observations raise the hypothesis that metformin could simultaneously address epithelial dysfunction, oxidative stress, and aberrant immune signaling in UC; however, translation of this multi-targeted profile to the clinical setting has not yet been fully demonstrated.

***Immunological pathway modulation:*** T cell differentiation represents a second mechanistic axis through which metformin has been proposed, on the basis of preclinical evidence, to exert anti-inflammatory effects. In murine models, metformin was found to suppress pathogenic γδT17 cells and interferon-γ-producing lamina propria CD4+ T cells through inhibition of mTOR/RORγt signaling [17]. Experimental data also suggest that metformin may enhance regulatory T cell (Treg) differentiation and promote interleukin-10 (IL-10) production while decreasing pro-inflammatory cytokines including IL-17, IL-6, and TNF-α in animal models [13]. Whether these immune-modulatory effects occur in human UC, and whether they are sufficient to restore the Th17/Treg balance clinically, remains uncertain. A 16-day murine experiment reported that metformin reduced IBD severity by suppressing phosphorylated STAT3 and IL-17 expression [13]; direct evidence for analogous mechanisms in human mucosal tissue is currently limited.

***Epithelial barrier integrity and tight junction preservation:*** Experimental models suggest that metformin may increase the expression of tight junction proteins including occludin, ZO-1, claudin-1, and tight junction protein-1, with these molecular changes associated with enhanced transepithelial electrical resistance and reduced FITC-dextran hyperpermeability in vitro—objective measures of epithelial barrier function [14]. These barrier-protective effects appear to depend, at least in part, on AMPK1 activation, as demonstrated by experiments in which AMPK1-silenced intestinal epithelial cell monolayers lost the capacity to maintain barrier integrity following dextran sulfate sodium (DSS) challenge [14]. In DSS-induced-colitis animal models, metformin administration was associated with prevention of body weight loss, inhibition of colon shrinkage, and preservation of colonic histological architecture [18]. The proposed mechanism underlying these barrier-protective effects involves suppression of JNK activation through an AMPK1-dependent pathway [14]. Although these findings are mechanistically compelling, it is important to emphasize that they were obtained in animal and cell systems, and it remains unclear to what extent they reflect the pathophysiology of human UC. Studies in intestinal epithelial cell-specific AMPK-knockout mice showed that AMPK deletion significantly delays wound healing and epithelial regeneration following acute colonic injury [18,19]. Notably, metformin administration during the recovery phase was reported to accelerate epithelial regeneration in both wild-type and AMPK-knockout mice [18], suggesting the existence of AMPK-independent protective pathways; the clinical relevance of this finding in human disease has not yet been established.

***Microbiota-mediated effects—restoration of dysbiosis:*** Additional preclinical work suggests that metformin may suppress certain pathogenic bacterial populations, such as *Shigella* and *Escherichia coli*, while potentially increasing the relative abundance of *Lactobacillus* spp., *Bacteroides*, and *Akkermansia muciniphila* in experimental models [12,20,21,22,23]. Whether this microbial remodeling is reproducible and clinically meaningful in human UC remains to be established. In animal studies, metformin-induced enrichment of *Akkermansia muciniphila* was associated with reduced colonic inflammation and increased mucin-2 expression, leading the authors to propose that this bacterium may contribute to the observed anti-inflammatory effects [24]; however, causality in a human context has not been demonstrated. Preclinical data further suggest that increased synthesis of short-chain fatty acids (SCFAs), particularly butyrate, may represent a microbiota-mediated effect of metformin [13], with SCFA-producing bacteria potentially reinforcing intestinal barrier integrity through G-protein-coupled receptor signaling and histone deacetylase inhibition in experimental models. Additionally, metformin-induced microbiota alterations have been reported in preclinical settings to increase TLR1 and TLR4 expression and promote glucagon-like peptide-1 (GLP-1) signaling [24], although human confirmatory data are lacking. Cecal microbiota transplantation experiments suggest that metformin-modified microbiota can reduce IL-17 expression in recipient animals [13]; the translational significance of this finding for human IBD treatment remains speculative at this stage.

***Intestinal fibrosis prevention and chronic complication management:*** Intestinal fibrosis is a recognized chronic complication of UC that may develop despite conventional anti-inflammatory treatment [25,26,27,28]. In animal models, metformin administration was reported to attenuate colitis-associated intestinal fibrosis through inhibition of the TGF-β1/Smad3 signaling cascade [29]. Both preventive and therapeutic regimens in TNBS- and DSS-induced chronic colitis models were associated with reduced intestinal fibrosis, mediated by inhibition of Smad3 phosphorylation and prevention of its nuclear translocation. Consistent with these in vivo findings, in vitro data indicate that metformin may reduce fibroblast proliferation, suppress extracellular matrix protein production, and decrease collagen synthesis in human colon fibroblast cell lines and primary human intestinal fibroblasts treated with TGF-β1 [30]. While these preclinical observations are of potential interest, it must be underscored that no clinical evidence currently exists demonstrating that metformin prevents or reverses intestinal fibrosis in humans with UC. These findings support a multifaceted, but still largely experimental, model in which metformin may target epithelial, immune, microbial, and fibrotic pathways; rigorous clinical validation of this model is needed.

## 4. Biological Mechanisms Between Metabolic Alterations and UC

Metabolic imbalance has emerged as a central trigger for UC pathology: reduced synthesis of short-chain fatty acids (SCFAs) compromises intestinal barrier function and immunomodulatory capacity while dysregulated bile acid metabolism and aberrant tryptophan pathways further disrupt epithelial integrity and activate and self-reinforce the inflammatory loop. Multiomics and proteomic studies consistently show downregulation of oxidative phosphorylation, β oxidation, glycolysis, and TCA cycloenzymes in UC mucosa, indicating mitochondrial energy failure and impaired antioxidant defenses that compromise epithelial barrier function and promote chronic inflammation [31,32,33,34]. Reduced colonic NAD^+^ with accumulation of nicotinamide and ADP ribose further links mitochondrial dysfunction to inflammatory activity and altered immune cell infiltration, defining distinct NAD^+^-dependent UC subtypes [33,35]. Parallel metabolomic work highlights broad disturbances in lipid and organic acid metabolism, including reduced microbially derived secondary bile acids and other co-metabolites, which are normally critical for maintaining epithelial integrity and immunoregulatory signaling [36,37,38]. Gut microbiota–metabolite analyses demonstrate that dysbiotic communities reshape the fecal metabolome, enriching cytotoxic sulfides and bile acids while depleting health-promoting short- and medium-chain fatty acids, thereby increasing epithelial cell death, weakening barrier function, and skewing cytokine responses toward a pro-inflammatory profile [39,40]. Specific microbial taxa, such as *Veillonella parvula*, use nitrate- and lactate-dependent pathways to generate immunomodulatory tryptophan metabolites and can even inactivate thiopurine drugs, directly coupling metabolic outputs to both immune tone and therapeutic response [39]. Mendelian randomization and pathway analyses extend this concept to a broader immune–metabolic axis, showing that inflammatory chemokines and cytokine receptors influence UC risk partly through lipid intermediates and other circulating metabolites [37,41]. Together, these findings support a model in which mitochondrial and redox defects in the epithelium, disordered sugar and mannose metabolism, and microbiota-derived metabolic shifts (in SCFAs, bile acids, tryptophan catabolites, and other small molecules) converge to erode barrier integrity, reprogram mucosal immunity, and perpetuate the chronic colonic inflammation characteristic of UC [31,34,35,36,38,39,40,41,42,43].

## 5. Clinical Studies

Recent evidence from double-blind, randomized controlled trials (RCTs) demonstrates that metformin, when used as an adjunctive (add-on) therapy to standard treatments like 5-ASA (mesalamine), significantly improves clinical and endoscopic outcomes in mild-to-moderate UC, with significant reductions in disease activity indices and inflammatory biomarkers [44,45,46,47,48,49]. Patients receiving metformin (typically 500 mg twice daily) showed significantly higher rates of clinical remission and mucosal healing than placebo groups. Metformin therapy was associated with a marked decrease in C-reactive protein (CRP) and fecal calprotectin. In these trials, a dose of 500 mg b.i.d was successfully utilized; the drug was well tolerated in these UC cohorts, with no significant increase in adverse events compared with placebo.

While the recent RCTs provide a “proof of concept,” several limitations must be considered before widespread clinical adoption. Most current trials of metformin in UC are relatively small (often < 100 per group), focused largely on mild-to-moderate UC and have a short follow-up (typically between 12 and 24 weeks). Also, these findings are not fully consistent across all epidemiologic designs. Extensive observational data and Mendelian randomization analyses provide discordant insights into the role of metformin in IBD risk and long-term disease modification [47]. A Danish nationwide new user, active comparator cohort did not find evidence that metformin reduces the risk of older-onset IBD, reporting an adjusted odds ratio of 1.04 (95% CI 0.83–1.31) for UC [48]. Similarly, a Mendelian randomization study did not detect a statistically significant causal effect of genetically proxied metformin treatment on UC incidence, even though an inverse association was observed at the phenotypic level; the authors thus cautioned that restriction to European populations might limit generalizability [48]. On the other hand, several observational cohorts suggest that metformin exposure may favorably influence disease course or primary prevention in specific contexts. A propensity-matched analysis of UC patients with type 2 diabetes reported that metformin therapy was associated with a sustained reduction in the need for intravenous steroids over a three-year follow-up [49]. Likewise, a nationwide Taiwanese cohort of more than 360,000 individuals with type 2 diabetes found that “ever users” of metformin had a substantially lower risk of incident IBD, with a clear duration of response gradient (HR 0.57 for 26.0–58.3 months and 0.24 for >58.3 months of therapy) [50]. These apparently divergent findings likely reflect differences in study design (efficacy trials in established mild-to-moderate UC versus large pharmacoepidemiologic cohorts assessing incident disease), outcome definitions (short-term clinical and endoscopic response versus IBD onset), populations (younger UC cohorts vs. older-onset IBD; Asian vs. European ancestry), and residual confounding related to diabetes severity, comedications, and healthcare utilization.

Taken together, randomized trials currently provide the most direct evidence that metformin can improve disease activity when used as adjunctive therapy in mild-to-moderate UC, whereas population-based observational and genetic studies offer mixed signals about its role in primary prevention or causal protection against incident UC (Table 1).

## 6. Clinical Studies: Proposed Mechanisms of Action

Human mechanistic data on metformin in UC are considerably more limited and indirect than the preclinical evidence reviewed above. Randomized clinical trials in mild-to-moderate UC have primarily assessed clinical indices and standard circulating or fecal biomarkers. These trials consistently report reductions in disease activity scores, C-reactive protein, fecal calprotectin, and nitric oxide with adjunctive metformin compared with control arms and document correlations between biomarker changes and clinical improvement [44,45,46]. One randomized trial reported increased colonic ZO-1 gene expression and decreased STAT3 and ICAM-1 expression in mucosal biopsies from metformin-treated patients [46], providing some—albeit limited—translational support for barrier-preserving and anti-inflammatory actions in human tissue. An interventional study in female UC patients demonstrated that metformin, alone or in combination with indole-3-carbinol, was associated with improvements in colonic endoscopic scores and significant reductions in tissue catalase, TNF-α, TGF-β1, malondialdehyde, and myeloperoxidase [51]. These human observations are directionally consistent with the preclinical mechanistic framework but do not constitute proof of the underlying molecular pathways. Taken together, mechanistic conclusions should be framed cautiously. Current preclinical evidence from in vitro and animal studies suggests that metformin may modulate AMPK-dependent signaling, epithelial barrier function, immune cell differentiation, microbiota composition, and fibrogenesis through several interconnected pathways. However, available human data are limited to clinical and biomarker endpoints, with only sparse and indirect molecular evidence from tissue biopsies. The complex mechanistic network proposed from animal and cell studies has not yet been fully validated in humans. Accordingly, the translation of these preclinical findings to clinical practice requires further investigation through well-designed mechanistic substudies embedded in future randomized trials.

## 7. Metformin as an Antineoplastic Agent in Patients with UC

It is worth noting that the use of metformin seems to lower the risk of colorectal neoplasias, as shown by experimental animal studies [52,53] and observations in humans [54,55]. Recent evidence from a large nationwide cohort study suggests that metformin use in IBD patients, a high-risk group, may offer substantial protection against colorectal cancer. Using Taiwan’s National Health Insurance Research Database linked to the Taiwan Cancer Registry, the researchers followed over 240,000 adults who were newly diagnosed with IBD between 2008 and 2019. In patients with concurrent IBD and diabetes, metformin users showed a significantly lower risk of colorectal cancer when compared with non-users (adjusted hazard ratio 0.44; 95% CI 0.29–0.68). This risk reduction was dose-dependent across cumulative exposure quartiles, reaching 67% at the highest exposure level. Interestingly, a daily intake of approximately 800 mg was associated with the lowest risk of cancer [56]. These results highlight the potential chemopreventive function of metformin in individuals with diabetes and IBD.

By contrast, the Korean cohort focused on newly diagnosed UC patients and evaluated overall and site-specific cancer risk versus the general population, along with potential chemopreventive effects of statins, metformin, and aspirin. In this setting, metformin showed no measurable chemopreventive benefit for cancer in UC, and the CRC incidence in UC was not higher than in the general Korean population, unlike many Western series. Thus, any incremental benefit of metformin may be difficult to detect against a relatively low baseline CRC risk [57]. Critically, the two cohorts differ in design, background of CRC risk and exposure intensity. Taken together, Taiwanese data support a potentially meaningful preventive and chemopreventive role for metformin in diabetic, higher-risk IBD populations, whereas Korean data caution against assuming a universal chemopreventive effect across all UC patients, especially where background cancer risk is already low (Table 2 and Table 3).

## 8. Ongoing Studies

Three ongoing clinical trials are exploring the potential therapeutic benefits of metformin in UC. Specifically, NCT04750135 broadly assesses the role of metformin as an adjuvant therapy. This randomized controlled study evaluates metformin tablets (1500 mg) administered daily for 8 weeks. This study aims to evaluate the efficacy and safety of metformin in the treatment of mild-to-moderately active UC. Disease activity will be measured using the Mayo score for UC activity. The calculation of the score requires patients to undergo colonoscopy at the start of the study and at week 8.

The completed NCT05553704 trial (data not yet published) focused on comparing the possible efficacy of metformin in patients with UC treated with mesalamine. The primary endpoint was the change in the partial Mayo score index, and the secondary endpoint was estimated by changes in serum IL-6 and TNF-alpha. Furthermore, the NCT05574387 trial analyzes metformin as an adjuvant therapy in patients with UC treated with mesalamine, and the primary endpoint of the study will be the improvement in HRQL through changes in the IBD questionnaire score and change in Mayo scores.

## 9. Safety Profile of Metformin and Its Use in the UC Population

Metformin demonstrates an excellent safety profile, with negligible serious adverse event rates documented across diverse patient populations over extended treatment periods [58,59,60]. The key issues regarding metformin tolerability are gastrointestinal disturbances, which may contribute to the reluctance to prescribe metformin in people with pre-existing gastrointestinal diseases. Diarrhea and nausea were reported by 20% and 25.5% of patients, respectively [61,62,63,64]. Multiple pathophysiological mechanisms have been proposed to explain the gastrointestinal effects of metformin. The occurrence of gastrointestinal adverse events seems to be a consequence of the kinetics and pharmacodynamics of metformin in enterocyte cells. Owing to its hydrophilic and cationic structure, metformin cannot cross the cell membrane via passive diffusion. Metformin’s bioavailability is 50–60% [65]. Transport from enterocytes into the systemic circulation is less efficient than that from the lumen to enterocytes, leading to metformin concentrations up to 300-fold higher in the small intestinal epithelium than in the bloodstream. For this reason, it has been postulated that part of the glucose-lowering action of metformin might take place in the intestine [66]. Lumen uptake by enterocytes is mediated by organic cation transporters (OCT1 and OCT3), plasma membrane monoamine transporter, and serotonin reuptake transporter (SERT). Genetic variations in organic cation transporter-1 (OCT1) seem to be involved in the pharmacokinetic mechanisms of metformin-induced gastrointestinal intolerance [67]. In the GoDARTS study, individuals with two reduced-function OCT1 alleles concomitantly treated with OCT1 inhibitors (tricyclic antidepressants, citalopram, proton-pump inhibitors, and spironolactone) exhibited more than a fourfold increase in the risk of metformin intolerance [67]. Serotonin released by mucosal enterochromaffin cells stimulates 5-hydroxytryptamine receptors number 3 (5-HT3) and 5-HT4 to enhance peristalsis and intestinal propulsion [68,69]. Metformin may cause gastrointestinal side effects by inhibiting SERT-mediated serotonin reuptake due to increased gastrointestinal motility induced by serotonin. Other mechanisms may explain the gastrointestinal disturbances associated with metformin. These include modifications in the intestinal microbiota composition [70], increased intracellular lactate concentrations subsequent to enhanced intestinal glucose utilization [71], elevated GLP-1 concentrations at the intestinal level, and increased intestinal water loss caused by the inhibition of ionic channels [72]. Metformin-related diarrhea could also result from the increased intestinal bile acid pool [73]. The absorption of bile acid in the ileum is an active process that involves the nuclear farnesoid X receptor (FXR), which can be phosphorylated by AMPK, resulting in a reduction in FXR transcription and increased bile salt presence in the intestinal lumen [74]. With respect to the microbiome, metformin treatment has been associated with an increased relative abundance of *Escherichia* spp. in people treated with metformin. *Escherichia* spp. have been associated with gas metabolism, and the prevalence of bloating and flatulence is 25% and 8%, respectively, in people treated with metformin [70]. The presence of *Akkermansia* increases with metformin treatment, contributing to the maintenance of intestinal barrier integrity; however, its specific role in the development of gastrointestinal side effects remains unclear. However, observational data specifically in IBD suggest that underlying UC does not itself amplify metformin-related GI symptoms, and large diabetic-IBD cohorts show sustained tolerability without excess serious events even beyond 58 months of exposure [49]. In randomized controlled trials of metformin as an adjunctive therapy for UC, the safety profile remained excellent, with minimal treatment-related adverse events requiring intervention [75].

For UC clinicians, several practical strategies can optimize safety. First, a cautious dose-titration approach (e.g., starting at 500 mg once or twice daily and up-titrating as tolerated) mirrors diabetes practice and may mitigate early GI complaints, particularly in patients with active diarrhea. Second, clinicians should recognize pharmacogenetic susceptibility: reduced-function OCT1 variants and concurrent OCT1 inhibitors markedly increase the risk of intolerance, so extra vigilance is warranted when tricyclic antidepressants, citalopram, proton-pump inhibitors or spironolactone are co-prescribed. Third, when layering metformin onto existing UC regimens, available data indicate no clinically relevant drug–drug interactions with mesalamine, corticosteroids, or immunomodulators and preserved efficacy and safety in diabetic patients on UC-specific therapies. Finally, because metformin is renally excreted and can rarely precipitate lactic acidosis in the setting of severe renal or hypoxic states (as recognized in diabetes guidelines), standard precautions—assessment of kidney function, avoidance in advanced renal failure, and temporary interruption during severe illness or major procedures—remain applicable to UC patients.

The safety profile of metformin in the context of UC warrants more-detailed consideration across several clinically relevant dimensions. Regarding formulation choice, extended-release and delayed-release preparations offer glycemic control equivalent to immediate-release tablets, while meaningfully reducing gastrointestinal adverse events and improving treatment adherence through once-daily dosing [76]. Long-term metformin use is consistently associated with reduced B12 levels, with deficiency prevalence ranging from roughly 7% to 40% depending on dose and duration; risk rises substantially at doses ≥1500–2000 mg/day and after ≥4–5 years of treatment, as metformin impairs intestinal B12 absorption by interfering with calcium-dependent binding of the intrinsic factor–B12 complex to ileal receptors [77].This concern is compounded in UC, where B12 status may already be compromised—particularly following subtotal colectomy or restorative proctocolectomy, in the setting of active disease with malabsorption, or through drug–nutrient interactions such as sulfasalazine-induced folate depletion—making periodic monitoring of B12, folate, and homocysteine especially warranted in this population. Additionally, UC patients face specific clinical scenarios that require practical guidance not always addressed in general metformin safety data: during episodes of significant dehydration from active flares or profuse diarrhea and in the peri-procedural period surrounding colonoscopy preparation, metformin should be used with caution or temporarily withheld, given the risk of reduced renal clearance and the theoretical concern for lactic acidosis in states of hemodynamic compromise [78]. Taken together, formulation selection of metformin, B12 surveillance strategies, and situation-specific guidance would substantially improve the clinicians’ management of UC patients taking metformin. Within these boundaries, current clinical trials in mild-to-moderate UC report an “excellent” safety profile, with very few treatment-related adverse events requiring discontinuation [79,80,81].

## 10. Conclusions

A balanced interpretation of the studies suggests that metformin is a promising repurposed adjunct in selected UC patients, particularly those already receiving mesalamine or living in settings with limited access to advanced therapies. Furthermore, metformin offers a particularly beneficial therapeutic opportunity to address intestinal inflammation and glycemic dysregulation through unified AMPK-dependent signaling cascades in the increasingly common subset of UC patients with type 2 diabetes mellitus. The prebiotic-like enrichment of beneficial gut bacteria by metformin also improves regulatory T cell differentiation and has long-lasting barrier-protective effects, which may lower relapse rates during remission maintenance phases and allow corticosteroid-sparing therapeutic approaches. However, the capacity of metformin to modify lifetime UC risk or long-term disease progression remains unclear, requiring further investigation on the underlying mechanisms by which metformin exerts its anti-inflammatory effects in UC management.

## Figures and Tables

**Table 1:** Clinical studies.

Study/Level of Evidence *	Design and Population	Dose and Duration	Primary Endpoints	Main UC Outcomes	Safety
**Binsaleh 2025** (High)	RCT, 60 adults with mild–moderate UC on mesalamine 1 g TID. Nondiabetic population.	Metformin 500 mg BID vs. no metformin; 6 months.	Change in disease activity index.	Greater reduction in clinical activity index (*p* = 0.0001); CRP, fecal calprotectin, and nitric oxide.	Well tolerated as an adjunct to mesalamine; no specific serious AEs (SAEs) reported.
**El-Haggar 2024** (High)	RCT in UC on mesalamine; mild–moderate activity. Nondiabetic population.	Metformin 500 mg BID vs. mesalamine alone; 6 months.	Endoscopic score; mucosal gene expression.	Higher endoscopic response (81.25%) and remission (50%); improved ZO-1, decreased STAT3, ICAM-1 gene expression.	No specific serious adverse events (SAEs) or dose-limiting toxicities.
**Molavi 2025** (High)	Double-blind, randomized, placebo-controlled pilot; 112 patients with mild–moderate UC. Nondiabetic population.	Metformin 1000 mg BID vs. placebo; 2 months.	UCDAI (global, rectal bleeding, stool frequency, endoscopy).	54% lower odds of higher UCDAI vs. placebo (OR 0.46; *p* = 0.044); improved physician assessment.	No safety alarms cited.
**Ahmed 2017** (Moderate)	Interventional study in female UC patients. Nondiabetic population.	Metformin alone or with indole-3-carbinol.	Endoscopic score; oxidative stress and cytokine markers.	Improved endoscopic scores, tissue CAT, TNF-α, TGF-β1, MDA, and MPO.	No safety alarms cited.
**Petrov 2025** (Moderate)	Propensity-matched cohort; 1323 UC + T2DM on metformin vs. comparators.	Usual antidiabetic dosing; follow-up to 3 years.	IBD outcomes (e.g., IV steroid requirement).	Metformin associated with decreased need for IV steroids in UC, sustained up to 3 years.	Long-term safety profile.
**Ali 2020** (Low–Moderate)	Retrospective single-center analysis; 52 UC and 45 CD.	Metformin use vs. non-use; 2-year observation.	Number of symptom flares.	Lower mean number of flares in metformin users with UC (0.33 vs. 1.3; *p* = 0.022).	No safety alarms cited.
**Allin 2022** (Moderate–Low)	Nested case–control (Danish nationwide cohort); 302,863 new users of glucose-lowering drugs.	“New-user” active comparator design.	Risk of older-onset IBD, CD, or UC.	No link found. Adjusted OR 1.04 (95% CI 0.83–1.31) for UC.	No safety alarms cited.
**Tseng 2021** (Moderate–High)	Nationwide cohort (Taiwan); >360,000 patients with type 2 diabetes.	Cumulative duration: <26 m, 26–58.3 m, and >58.3 m.	Primary prevention of new-onset IBD	Significant risk reduction. Dose–response seen; HR 0.24 for duration >58.3 months.	No specific adverse events reported.
**Liao 2025** (Moderate–High)	Mendelian randomization (European populations).	Metformin treatment (genetically instrumented).	Risk of ulcerative colitis (UC).	No significant causal relationship found.	No specific adverse events reported.

* Levels of evidence are defined as: high = randomized controlled study; moderate = well-designed cohort/interventional study; low–moderate = small or retrospective single-center study.

**Table 2:** Metformin as antineoplastic agent: evidence from animal studies.

Author(s)	Experimental Model	Biochemical Mechanisms	Key Findings
**Lai 2025**	Enterotoxigenic Bacteroides Fragilis (ETBF)/azoxymethane (AOM)/dextran sulfate sodium (DSS) mouse model. Low-dose metformin (125 mg/kg) or high-dose metformin (250 mg/kg) administered daily by gavage.	Suppression of TLR4/MyD88/NFκB/MAPK pathway, modulation of macrophage M2 polarization and increase in SCFA levels.	Metformin inhibits colorectal tumorigenesis.
**Wang 2025**	Azoxymethane combined with dextran sulfate sodium-treated BALB/C female mice. Metformin doses of 125 mg/kg, 250 mg/kg or 500 mg/kg administered daily by gavage.	Improvement of intestinal mucosal barrier function by affecting the expression of tight junction proteins in the intestinal mucosa, including occludin, ZO-1, and claudins.	Metformin reduces the number and diameter of CRC tumors induced by AOM/DSS compared with those in the control group.

**Table 3:** Metformin as antineoplastic agent: evidence from human studies.

Author(s)	Study Population	Key Findings	Clinical Significance
**Huang 2025**	240,000+ Taiwanese IBD patients (2008–2019)	Significant risk reduction for CRC (adjusted hazard ratio: 0.44). Dose-dependent reduction up to 67%.	Dose-dependent effect; reduction reached 67% at highest exposure; optimal dose ~800 mg/day in IBD/diabetes patients.
**Oh 2025**	35,189 Korean UC patients (2007–2020)	No measurable chemopreventive benefit of metformin for cancer in UC.	CRC incidence in this specific UC population was not higher than in the general population.
**Rokkas 2016**	Patients with type 2 diabetes	Metformin lowers the risk of CRC.	Metformin in patients with type 2 diabetes is associated with significantly lower risk of colon neoplasia.
**Xu 2024**	Patients with colon cancer	Meta-analysis showing better prognosis for those using metformin and statins.	Metformin may improve the prognosis of colon cancer.

## Data Availability

No new data were created or analyzed in this study. Data sharing is not applicable to this article.

## Abbreviations

The following abbreviations are used in this manuscript:

AMPK	adenosine monophosphate-activated protein kinase
CAT	tissue catalase
CD	Crohn’s disease
CHOP	C/EBP homologous protein
DSS	dextran sulfate sodium
ER	endoplasmic reticulum
FXR	farnesoid X receptor
GLP-1	glucagon-like peptide-1
GRP78	glucose-regulated protein 78
HT	hydroxytryptamine
IL	interleukin
IBD	inflammatory bowel disease
JNK	Jun N-terminal kinase
MDA	malondialdehyde
mTOR	mammalian target of rapamycin
MPO	myeloperoxidase
OCT	organic cation transporter
SCFAs	short-chain fatty acids
SERT	serotonin reuptake transporter
SPHK1/S1P	sphingosine kinase 1/sphingosine 1-phosphate
UC	ulcerative colitis
UCDAI	UC Disease Activity Index
TGF	transforming growth factor
TLR	toll-like receptor
Treg	regulatory T cell
ZO-1	zonula occludens-1

## References

[1] Gros B., Kaplan G.G. (2023). Ulcerative colitis in adults: A review. JAMA.

[2] Juhl A.E., Westfall M., Hebbelstrup Jensen B., Mirsepasi-Lauridsen H.C. (2025). Gut microbiota in IBD: The beneficial and adverse effects of diet and medication. Nutrients.

[3] Villanacci V., Del Sordo R., Parigi T.L., Leoncini G., Bassotti G. (2023). Inflammatory bowel diseases: Does one histological score fit all?. Diagnostics.

[4] Hracs L., Windsor J.W., Gorospe J., Cummings M., Coward S., Buie M.J., Quan J., Goddard Q., Caplan L., Markovinović A. (2025). Global evolution of inflammatory bowel disease across epidemiologic stages. Nature.

[5] Katsoula A., Paschos P., Malandris K., Kalogirou M.S., Haidich A.B., Giouleme O., Tsapas A. (2025). Systematic review and network meta-analysis: Evaluating the impact of advanced therapies for moderate-to-severe ulcerative colitis on health-related quality of life. J. Crohns Colitis.

[6] Moran G.W., Gordon M., Sinopolou V., Radford S.J., Darie A.M., Vuyyuru S.K., Alrubaiy L., Arebi N., Blackwell J., Butler T.D. (2025). British Society of Gastroenterology guidelines on inflammatory bowel disease in adults: 2025. Gut.

[7] Marafini I., Salvatori S., Fonsi A., Monteleone G. (2026). Optimizing drug positioning in IBD: Clinical predictors, biomarkers, and practical approaches to personalized therapy. Biomedicines.

[8] Maruthur N.M., Tseng E., Hutfless S., Wilson L.M., Suarez-Cuervo C., Berger Z., Chu Y., Iyoha E., Segal J.B., Bolen S. (2016). Diabetes medications as monotherapy or metformin-based combination therapy for type 2 diabetes: A systematic review and meta-analysis. Ann. Intern. Med..

[9] Drewe J., Foretz M., Krähenbühl S. (2026). Metformin-mechanisms of its glycemia-reducing effect. Pharmacol. Rev..

[10] Garber A.J., Duncan T.G., Goodman A.M., Mills D.J., Rohlf J.L. (1997). Efficacy of metformin in type II diabetes. Am. J. Med..

[11] Sun M., Liu H., Ju H., Chen H., Yang R., Yan D., Shen L., Cai A., Zhi Y., Xiao L. (2025). Metformin suppresses gammadelta T17 cell differentiation alleviating DSS-induced colitis. Immunol. Res..

[12] Lee H., Ko G. (2014). Effect of metformin on metabolic improvement and gut microbiota. Appl. Environ. Microbiol..

[13] Lee S.Y., Lee S.H., Yang E.J., Kim E.K., Kim J.K., Shin D.Y., Cho M.L. (2015). Metformin ameliorates inflammatory bowel disease by suppression of the STAT3 signaling pathway and regulation of the between Th17/Treg balance. PLoS ONE.

[14] Deng J., Zeng L., Lai X., Li J., Liu L., Lin Q., Chen Y. (2018). Metformin protects against intestinal barrier dysfunction via AMPKα1-dependent inhibition of JNK signalling activation. J. Cell. Mol. Med..

[15] Di Fusco D., Dinallo V., Monteleone I., Laudisi F., Marafini I., Franzè E., Di Grazia A., Dwairi R., Colantoni A., Ortenzi A. (2018). Metformin inhibits inflammatory signals in the gut by controlling AMPK and p38 MAP kinase activation. Clin. Sci..

[16] Wang J., Chen C., Ren Y., Zhou X., Yu S. (2021). Metformin alleviates intestinal epithelial barrier damage by inhibiting endoplasmic reticulum stress-induced cell apoptosis in colitis cell model. J. Zhejiang Univ. Med. Sci.

[17] Youssef M.E., Abd El-Fattah E.E., Abdelhamid A.M., Eissa H., El-Ahwany E., Amin N.A., Hetta H.F., Mahmoud M.H., Batiha G.E., Gobba N. (2021). Interference with the AMPKα/mTOR/NLRP3 signaling and the IL-23/IL-17 axis effectively protects against the dextran sulfate sodium intoxication inrRats: A new paradigm in empagliflozin and metformin reprofiling for the management of ulcerative colitis. Front. Pharmacol..

[18] Chen L., Wang J., You Q., He S., Meng Q., Gao J., Wu X., Shen Y., Sun Y., Wu X. (2018). Activating AMPK to restore tight junction assembly in intestinal epithelium and to attenuate experimental colitis by metformin. Front. Pharmacol..

[19] Olivier S., Diounou H., Pochard C., Frechin L., Durieu E., Foretz M., Neunlist M., Rolli-Derkinderen M., Viollet B. (2022). Intestinal epithelial AMPK deficiency causes delayed colonic epithelial repair in DSS-induced colitis. Cells.

[20] Shin N.R., Lee J.C., Lee H.Y., Kim M.S., Whon T.W., Lee M.S., Bae J.W. (2014). An increase in the *Akkermansia* spp. population induced by metformin treatment improves glucose homeostasis in diet-induced obese mice. Gut.

[21] Wang Y., Jia X., Cong B. (2024). Advances in the mechanism of metformin with wide-ranging effects on regulation of the intestinal microbiota. Front. Microbiol..

[22] Caturano A., Nilo D., Nilo R., Sircana M.C., Erul E., Zielińska K., Russo V., Santonastaso E., Sasso F.C. (2026). Old drug, new science: Metformin and the future of pharmaceutics. Pharmaceutics.

[23] Ke H., Li F., Deng W., Li Z., Wang S., Lv P., Chen Y. (2021). Metformin exerts anti-inflammatory and mucus barrier protective effects by enriching *Akkermansia muciniphila* in mice with ulcerative colitis. Front. Pharmacol..

[24] Kim M.H., Jee J.H., Park S., Lee M.S., Kim K.W., Lee M.K. (2014). Metformin enhances glucagon-like peptide 1 via cooperation between insulin and Wnt signaling. J. Endocrinol..

[25] Antonelli E., Giuliano V., Casella G., Villanacci V., Baldini V., Baldoni M., Morelli O., Bassotti G. (2011). Ultrasonographic assessment of colonic wall in moderate-severe ulcerative colitis: Comparison with endoscopic findings. Dig. Liver Dis..

[26] Ippolito C., Colucci R., Segnani C., Errede M., Girolamo F., Virgintino D., Dolfi A., Tirotta E., Buccianti P., Di Candio G. (2016). Fibrotic and vascular remodelling of colonic wall in patients with active ulcerative colitis. J. Crohn’s Colitis.

[27] Krugliak Cleveland N., Torres J., Rubin D.T. (2022). What does disease progression look like in ulcerative colitis, and how might it be prevented?. Gastroenterology.

[28] Villanacci V., Maconi G., Laschi L., Bassotti G. (2025). Diagnosing ulcerative colitis: Should we go beyond the surface?. J. Clin. Med..

[29] Liu X., Sun Z., Wang H. (2021). Metformin alleviates experimental colitis in mice by up-regulating TGF-β signaling. Biotech. Histochem..

[30] Wang Y., Wang Z., Yang H., Chen S., Zheng D., Liu X., Jiang Q., Chen Y. (2022). Metformin ameliorates chronic colitis-related intestinal fibrosis via inhibiting TGF-β1/Smad3 signaling. Front. Pharmacol..

[31] Noh J.Y., Farhataziz N., Kinter M.T., Yan X., Sun Y. (2024). Colonic Dysregulation of Major Metabolic Pathways in Experimental Ulcerative Colitis. Metabolites.

[32] Wang H., Xie Q., Xie Y., Luo W. (2025). Comprehensive proteomic profiling of intestinal tissues in patients with ulcerative colitis. Front. Med..

[33] Kang Y.H., Tucker S.A., Quevedo S.F., Inal A., Korzenik J.R., Haigis M.C. (2022). Metabolic analyses reveal dysregulated NAD+ metabolism and altered mitochondrial state in ulcerative colitis. PLoS ONE.

[34] Chen T., Tao Y., Wang Q., Pei Y., Zhao Z., Yang W., Lu Y. (2024). Utilizing an integrated bioinformatics and machine learning approach to uncover biomarkers linking ulcerative colitis to purine metabolism-related genes. Heliyon.

[35] Mu M., Xu Q., Hao Q., Li X., Wu Z., Zhang Q., Liu S., Man X., Xiao L., Zou Y. (2025). Multiomics Analysis of *Bacteroides cellulosilyticus* Anticolitis via Gut Microbiota Metabolite-Mediated PI3K-Akt Signaling Pathway. J. Agric. Food Chem..

[36] Liu M., Guo S., Wang L. (2024). Systematic review of metabolomic alterations in ulcerative colitis: Unveiling key metabolic signatures and pathways. Ther. Adv. Gastroenterol..

[37] Gallagher K., Catesson A., Griffin J.L., Holmes E., Williams H.R.T. (2021). Metabolomic Analysis in Inflammatory Bowel Disease: A Systematic Review. J. Crohns Colitis.

[38] Hyun C.K. (2021). Molecular and Pathophysiological Links between Metabolic Disorders and Inflammatory Bowel Diseases. Int. J. Mol. Sci..

[39] Schirmer M., Stražar M., Avila-Pacheco J., Rojas-Tapias D.F., Brown E.M., Temple E., Deik A., Bullock K., Jeanfavre S., Pierce K. (2024). Linking microbial genes to plasma and stool metabolites uncovers host-microbial interactions underlying ulcerative colitis disease course. Cell Host Microbe.

[40] Poppe J., Boesmans L., Vieira-Silva S., Deroover L., Tito R., Vandeputte D., Vandermeulen G., De Preter V., Raes J., Vermeire S. (2024). Differential contributions of the gut microbiota and metabolome to pathomechanisms in ulcerative colitis: An in vitro analysis. Gut Microbes.

[41] Cai M., Liu Y., Tao H., Tang L., Zhao L., Lan W., Liu X., Sheng Z., Peng Y., Sun W. (2025). Mendelian randomization study: Metabolites as mediators of inflammatory factors in ulcerative colitis. Medicine.

[42] Sankarasubramanian J., Ahmad R., Avuthu N., Singh A.B., Guda C. (2020). Gut Microbiota and Metabolic Specificity in Ulcerative Colitis and Crohn’s Disease. Front. Med..

[43] Liu Y., Jiang H., Gu Y., Li Y., Ding X. (2025). Multiple Comprehensive Analyses Identify the Protective Role and Diagnostic Signature of Mannose Metabolism in Ulcerative Colitis. Int. J. Mol. Sci..

[44] Binsaleh A.Y., El-Haggar S.M., Hegazy S.K., Maher M.M., Bahgat M.M., Elmasry T.A., Alrubia S., Alsegiani A.S., Eldesoqui M., Bahaa M.M. (2025). The adjunctive role of metformin in patients with mild to moderate ulcerative colitis: A randomized controlled study. Front. Pharmacol..

[45] Molavi M., Amin B., Sahebkar M. (2025). Metformin as a potential adjunct in the treatment of mild-to-moderate ulcerative colitis: A double-blind, randomized, placebo-controlled pilot study. Intern. Emerg. Med..

[46] El-Haggar S.M., Hegazy S.K., Maher M.M., Bahgat M.M., Bahaa M.M. (2024). Repurposing metformin as adjuvant therapy in patients with ulcerative colitis treated with mesalamine: A randomized controlled double-blinded study. Int. Immunopharmacol..

[47] Allin K.H., Jensen C.B., Jacobsen R.K., Jess T. (2022). Metformin use is not associated with reduced risk of older onset inflammatory bowel disease: A Danish nationwide population-based study. J. Gastroenterol..

[48] Liao Z., Su C., Li J., Liu J. (2025). Causal association of metformin treatment with diverse immune-mediated inflammatory diseases: A Mendelian randomization analysis. Medicine.

[49] Petrov J.C., Desai A.A., Kochhar G.S., Crosby S.K., Kinnucan J.A., Picco M.F., Hashash J.G., Farraye F.A. (2025). Metformin is associated with improved inflammatory bowel disease outcomes in patients with type 2 diabetes mellitus: A propensity-matched cohort study. Inflamm. Bowel Dis..

[50] Tseng C.H. (2021). Metformin use is associated with a lower risk of inflammatory bowel disease in patients with type 2 diabetes mellitus. J. Crohns Colitis.

[51] Kabel A.M., Omar M.S., Alotaibi S.N., Baali M.H. (2017). Effect of Indole-3-carbinol and/or metformin on female patients with ulcerative colitis (premalignant condition): Role of oxidative stress, apoptosis and proinflammatory cytokines. J. Cancer Res. Treat..

[52] Lai X., Liu B., Wan Y., Zhou P., Li W., Hu W., Gong W. (2025). Metformin alleviates colitis-associated colorectal cancer via inhibition of the TLR4/MyD88/NFκB/MAPK pathway and macrophage M2 polarization. Int. Immunopharmacol..

[53] Wang R., Xie L., Jiang P., Hou Y., Li D., Wang W. (2025). Metformin may improve intestinal mucosal barrier function and help prevent and reverse colorectal cancer in mice. J. Cancer.

[54] Rokkas T., Portincasa P. (2016). Colon neoplasia in patients with type 2 diabetes on metformin: A meta-analysis. Eur. J. Intern. Med..

[55] Xu Y., Che H., Liu J., Ye P. (2024). Association of metformin and statin uses with the prognosis of colon cancer: A meta-analysis. Eur. J. Cancer Prev..

[56] Huang Y.J., Lin J.A., Chen W.M., Shia B.C., Wu S.Y. (2025). Metformin use and risk of colorectal cancer in patients with inflammatory bowel disease: A nationwide, population-based cohort study. J. Natl. Cancer Inst..

[57] Oh E.H., Kim Y.J., Kim M., Park S.H., Kim T.O., Park S.H. (2025). Risk of malignancies and chemopreventive effect of statin, metformin, and aspirin in Korean patients with ulcerative colitis: A nationwide population-based study. Intest. Res..

[58] Sanchez-Rangel E., Inzucchi S.E. (2017). Metformin: Clinical use in type 2 diabetes. Diabetologia.

[59] Nishimura R., Taniguchi M., Takeshima T., Iwasaki K. (2022). Efficacy and safety of metformin versus the other oral antidiabetic drugs in Japanese type 2 diabetes patients: A network meta-analysis. Adv. Ther..

[60] Bailey C.J. (2024). Metformin: Therapeutic profile in the treatment of type 2 diabetes. Diabetes Obes. Metab..

[61] Foss M.T., Clement K.D. (2001). Metformin as a cause of late-onset chronic diarrhea. Pharmacotherapy.

[62] Strack T. (2008). Metformin: A review. Drugs Today.

[63] Stein S.A., Lamos E.M., Davis S.N. (2013). A review of the efficacy and safety of oral antidiabetic drugs. Expert Opin. Drug Saf..

[64] McCreight L.J., Bailey C.J., Pearson E.R. (2016). Metformin and the gastrointestinal tract. Diabetologia.

[65] Scheen A.J. (1996). Clinical pharmacokinetics of metformin. Clin. Pharmacokinet..

[66] Buse J.B., DeFronzo R.A., Rosenstock J., Kim T., Burns C., Skare S., Baron A., Fineman M. (2016). The primary glucose-lowering effect of metformin resides in the gut, not the circulation: Results from short-term pharmacokinetic and 12-week dose-ranging studies. Diabetes Care.

[67] Dujic T., Zhou K., Donnelly L.A., Tavendale R., Palmer C.N., Pearson E.R. (2015). Association of organic cation transporter 1 with intolerance to metformin in type 2 diabetes: A GoDARTS study. Diabetes.

[68] Stasi C., Bellini M., Bassotti G., Blandizzi C., Milani S. (2014). Serotonin receptors and their role in the pathophysiology and therapy of irritable bowel syndrome. Tech. Coloproctology.

[69] Spencer N.J., Keating D.J. (2025). Role of 5-HT in the enteric nervous system and enteroendocrine cells. Br. J. Pharmacol..

[70] Huang Y., Lou X., Jiang C., Ji X., Tao X., Sun J., Bao Z. (2022). Gut microbiota is correlated with gastrointestinal adverse events of metformin in patients with type 2 diabetes. Front. Endocrinol..

[71] Bailey C.J., Wilcock C., Scarpello J.H.B. (2008). Metformin and the intestine. Diabetologia.

[72] Han Y., Yun C.C. (2022). Metformin inhibits Na+/H+ exchanger NHE3 resulting in intestinal water loss. Front. Physiol..

[73] Carter D., Howlett H.C., Wiernsperger N.F., Bailey C.J. (2003). Differential effects of metformin on bile salt absorption from the jejunum and ileum. Diabetes Obes. Metab..

[74] Sadeeqa S., Fatima M., Latif S., Afzal H., Nazir S.U.R., Saeed H. (2019). Prevalence of metformin-induced gastrointestinal problems. Acta Pol. Pharm.–Drug Res..

[75] Niknejad A., Hosseini Y., Nazari Manesh M.A., Navabakhsh M., Peyrovinasab A., Mavaddat H., Miri M.S., Hosseini M., Büsselberg D., Abdolghaffari A.H. (2025). Therapeutic impacts of oral anti-diabetic drugs on Inflammatory Bowel Disease (IBD), a comprehensive review of clinical and preclinical studies. Naunyn Schmiedebergs Arch. Pharmacol..

[76] Tarry-Adkins J.L., Grant I.D., Ozanne S.E., Reynolds R.M., Aiken C.E. (2021). Efficacy and Side Effect Profile of Different Formulations of Metformin: A Systematic Review and Meta-Analysis. Diabetes Ther..

[77] Atkinson M., Gharti P., Min T. (2024). Metformin Use and Vitamin B12 Deficiency in People with Type 2 Diabetes. What Are the Risk Factors? A Mini-systematic Review. touchREV. Endocrinol..

[78] Chirila A., Nguyen M.E., Tinmouth J., Halperin I.J. (2022). Preparing for Colonoscopy in People with Diabetes: A Review with Suggestions for Clinical Practice. J. Can. Assoc. Gastroenterol..

[79] Wanchaitanawong W., Thinrungroj N., Chattipakorn S.C., Chattipakorn N., Shinlapawittayatorn K. (2022). Repurposing metformin as a potential treatment for inflammatory bowel disease: Evidence from cell to the clinic. Int. Immunopharmacol..

[80] Shinzaki S., Sato T., Fukui H. (2023). Antidiabetic drugs for IBD: A long but promising road ahead for drug repositioning to target intestinal inflammation. J. Gastroenterol..

[81] Antonelli E., Bassotti G. (2026). Repurposing metformin: Another possible bullet to target cheaply ulcerative colitis. Intern. Emerg. Med..

